# Highly private blockchain-based management system for digital COVID-19 certificates

**DOI:** 10.1007/s10207-022-00598-3

**Published:** 2022-07-29

**Authors:** Rosa Pericàs-Gornals, Macià Mut-Puigserver, M. Magdalena Payeras-Capellà

**Affiliations:** grid.9563.9 0000 0001 1940 4767 Dpt. de Ciències Matemàtiques i Informàtica, Universitat de les Illes Balears, 07122 Palma, Spain

**Keywords:** Health data, Blockchain, Privacy, Smart contract, Re-encryption, Self-sovereignty

## Abstract

As a result of the declaration of the COVID-19 pandemic, several proposals of blockchain-based solutions for digital COVID-19 certificates have been presented. Considering that health data have high privacy requirements, a health data management system must fulfil several strict privacy and security requirements. On the one hand, confidentiality of the medical data must be assured, being the data owner (the patient) the actor that maintain control over the privacy of their certificates. On the other hand, the entities involved in the generation and validation of certificates must be supervised by a regulatory authority. This set of requirements are generally not achieved together in previous proposals. Moreover, it is required that a digital COVID-19 certificate management protocol provides an easy verification process and also strongly avoid the risk of forgery. In this paper we present the design and implementation of a protocol to manage digital COVID-19 certificates where individual users decide how to share their private data in a hierarchical system. In order to achieve this, we put together two different technologies: the use of a proxy re-encryption (PRE) service in conjunction with a blockchain-based protocol. Additionally, our protocol introduces an authority to control and regulate the centers that can generate digital COVID-19 certificates and offers two kinds of validation of certificates for registered and non-registered verification entities. Therefore, the paper achieves all the requirements, that is, data sovereignty, high privacy, forgery avoidance, regulation of entities, security and easy verification.

## Introduction

COVID-19 is the disease caused by the coronavirus known as SARS-CoV2, a type of virus that causes a variety of illnesses ranging from a common mild cold to more serious illnesses such as pneumonia, Middle East respiratory syndrome (MERS) and severe acute respiratory syndrome (SARS), severe illnesses that can even cause the death to those who suffer from them.

In addition to being highly transmissible, it has led to the introduction of strong global restrictions, such as strict confinements of the population to reduce the effects and avoid the collapse of the hospitals. Specifically, as of 14 September 2021, 225.2 million cases have been reported worldwide together with 4.6 million deaths.^1^

As a consequence of the strong impact of the coronavirus crisis on the health sector, other areas of the economy have also been affected. Concretely the World Bank [1] indicated that the world economy in year 2020 would shrink by 5.2% and were expected to be the worst recession since the Second World War. Finally this predicted data was not reached, but there was a contraction of 4.3% of the world economy, which is enough to place it in the fourth position of the last 150 years [2].

In order to be able to reduce the effects of COVID-19 as soon as possible and to be able to return to a normal live, it is necessary to keep track of the population, recording whether a person is infected with COVID-19 at any given time, whether they have been infected and developed antibodies and, finally, whether they have been vaccinated against COVID-19. It is therefore proposed to use Digital COVID-19 Certificates [3] where all these cases can be easily checked.

However, it is important to take in mind that in order to obtain the expected result, the Digital COVID-19 Certificate must be difficult or impossible to forge. Nowadays, thousands of cases of fake Digital COVID-19 Certificates have been already reported, detected in different countries where the use of the certificate is already mandatory. For this reason, the number of people who can generate Digital COVID-19 certificates must be very restricted, otherwise forgery is facilitated. For example, in Munich a case is investigated where an employee of a pharmacy sold fraudulent vaccination certificates by simply gaining unauthorised access to the pharmacy’s network [4].

In addition, due to certificate falsification, there have already been cases of dead of infected people with COVID-19 who, upon arrival at the hospital, claim to be vaccinated. In consequence, they were not given a strong dose of medication, and when was too late, it was discovered that the person was not vaccinated [5, 6]. EU certificates, for instance, are easily verifiable by all countries, but each one has its own regulations and issuing controls, which makes them susceptible to forgery.

Such digital certificates contain health data, data that today have a high risk. The current rate of health data breaches has led to the need for privacy protection regulations such as Europe’s General Data Protection Regulation (GDPR). It requires the storage and sharing of your data in a secure and privacy-preserving manner, in order to enable the imposition of severe penalties for breaching these laws [7, 8].

Therefore, it is necessary to introduce immutability of data, i.e. to ensure that data cannot be modified once it is created. At the same time, it is necessary to introduce the authenticity of the entities in charge of publishing the data in the system. Objectives achieved in the proposed protocol with the use of blockchain technology and a regulatory authority that controls the entities that introduce the data into the system.

In the present work, blockchain technology provides the set of features necessary for the implementation of a decentralised application (DApp) to perform the management and certification of healthcare documentation, as well as providing the immutability of the healthcare data [9–11].

To achieve the necessary confidentiality of healthcare data we have introduced a threshold Proxy Re-Encryption (PRE) service, which provides the desired cryptography features to the data transmitted between the owning user and the requesting entities. By using elliptic curve cryptography, it is able to transform a ciphertext computed under Alice’s public key into one that can be opened by Bob’s secret key, but without giving him her secret key. Therefore, it achieves self-management of the data by its owners, introducing self-sovereign identity (SSI), so that the owners have full control of their information [12].

The paper is organised in the following sections. After this introduction, we present the state of the art on the objective of this paper, in Sect. 2. The contribution of this research is described in Sect. 3, followed by Sect. 4, where the background technologies of the implementation are outlined. Then, the system overview is depicted in Sect. 5, while Sect. 6 contains the description of the protocol designed for digital COVID-19 certificates management using blockchain technology. Section 7 describes the implementation and the smart contracts of the protocol, including the implementation of the proxy re-encryption service. Then, a security and privacy analysis of the system and the performance analysis of the protocol are included in Sects. 8 and 9, respectively. At last, the conclusions and future work are enumerated in Sect. 10.

## State of the art

In order to correctly implement digital COVID-19 certificates it is necessary to deal with medical data, and therefore, current solutions follow the same path as implementations that deal with medical data. The reason why blockchain technology has become a potential solution to meet the main requirements is that it provides a decentralised and tamper-proof network, in addition to enabling transparency of information. Therefore, it allows preventing the deletion of data and increasing the possibility of detection of malicious users [13].

The first proposal we found for a decentralised approach to meet the needs of medical record sharing applications was the MedRec application [14]. It provides patients a complete and immutable record and an easy access to their medical information across all providers and treatment centres. Using smart contracts, it registers client–provider relationships and generates permissions to view the patient’s medical information [15].

Nowadays, there are several proposals related to Digital COVID-19 certificates, taking advantage of blockchain technology. A DHP Framework using a private blockchain and Proof of Authority is proposed by Angelopoulos et al. in [16], where the Health Service Authorities are the ones responsible of the blockchain blocks generation. Therefore, the system loses most of the strong features of decentralised applications using the blockchain technology. In addition, as the authors point out, it does not provide full anonymity of end users, high security against forgery, as well as allowing the blockchain members to examine data without an access permission. Furthermore, the DHP will only be issued if the test results indicate that they are risk free. The paper does not provide any implementation details nor performance evaluation.

The VacciFi framework [17] is a GDPR compliant vaccination passport, based on a permissioned blockchain where it stores a verifiable hash of the vaccination passports information, saved in an off-chain storage system. Authors indicate that the off-chain storage uses the necessary access mechanisms to provide the security required, but does not provide any information about the security techniques used.

In [18], Odoom et al. propose a protocol solution for Covid-19 test results and vaccination using the blockchain technology and the IPFS system for encrypted results storage. The verifiers only check the owner signature over the data and obtain the test or vaccination status, information stored in the blockchain without encryption. Therefore, any user will be able to access all the test results of an identified Ethereum address.

Also, actually there are proposals that follows the World Wide Web Consortium standard “Verifiable Credentials”, like in [19], where the blockchain is used in conjunction with the descentralised personal data Solid Pod technology, which involve the user spending part of their device’s storage to save their certificates or relying on a cloud provider. Furthermore, the proposed system by Eisenstadt et. al does not provide neither a verifiers system registry nor any regulatory authority to manage and check the verifiers. Hasan et al.’s work [20] implement digital medical passports and immunity certificates using the Ethereum blockchain with IPFS and a proxy re-encryption scheme, obtaining a high descentralised application. However, it does not provide technical details on the proxy re-encryption scheme developed, only presents the smart contracts’s algorithms. In addition, this solution does not present any control over the verification entities, as other proposals, like NovidChain [21], where the W3C VC standard is used over an Ethereum private permissioned blockchain implementation named uPort and the IPFS storage.

In summary, there are several proposals that obtain solutions for the management of COVID-19 certificates, using innovative and secure technologies, but in general they do not present the technical details of a development and the cryptographic techniques used neither a security analysis that prove the fulfilment of the requirements, so the complete privacy and security of the solutions are not proved.

As a conclusion of the state-of-the-art analysis, to obtain a powerful solution it is important to provide the COVID-19 certificate management system with data integrity, immutability, privacy and confidentiality using cryptographic techniques, while providing self-sovereign identity to data owners and facilitating the verification process. Moreover, the use of a decentralised approach and the introduction of a regulatory authority to check the different registered actors would be desirable to avoid the possibility of forgery.

## Contribution

As explained in previous Sect. 2, state of the art, nowadays there are many proposals trying to achieve a solution against COVID-19, using innovative and secure technologies but they do not take into account the high privacy requirements and the data sovereignty. In our opinion a good digital COVID-19 certificate proposal must take into account these requirements and achieve an easy verification and a low forgery chance. So for that, our proposal achieves the following aspects that define our contribution:

- **Supervision of issuers and verifiers** The proposed protocol provides a method to control the entities that can issue certificates, avoiding forgery. The system also provides two verification subprotocols for both registered and a non-registered entities.
- **Encryption of all the digital COVID-19 certificates data** The encryption and re-encryption procedures used in the protocol make it a high privacy preserving protocol. There is no need to publish the certificates in the blockchain, but the procedure allows to verify all the data included in them.
- **Data sovereignty** The owners of the certificates (patients) are the actors in charge of the management of the consent to access their certificates. The users can check their certificates and also decide with whom they want to share them. Only the entities authorised by the owners will be able to decrypt the certificate thanks to the re-encryption scheme.
- **Implementation** The paper introduces not only the design, but also the implementation of the protocol, proving its viability. The paper includes some technical details of the DApp: the proxy re-encryption scheme implemented and the different smart contracts defined. The proposed protocol has been analysed in terms of security and privacy. Moreover, the paper includes a vulnerability analysis of the smart contracts. For more details, the implementation of the protocol can be found in our organisation GitHub account.^2^

## Background

### Blockchain

Blockchain is a digital, descentralised and distributed ledger, whose architecture has been developed from Satochi Nakamoto’s article published in 2008. The blockchain contains cryptographically signed transactions grouped in blocks and each block contains the transactions data, the timestamp, the cryptographic hash of the block (identificator of the block) and the cryptographic hash of the previous block, i.e. is linked with the previous one, generating a kind of chain.

The blockchain nodes are the ones responsible to introduce the new blocks, but first a common consensus algorithm is needed, to decide which blocks have to be added first. Therefore, from the different implementations of blockchain, different consensus algorithms have been determined such as proof of work, proof of stake and proof of authority, between others. Therefore, by updating the state of the network at every node, the immutability of the transactions is achieved.

Blockchain technology is creating a revolution in the field of secure data sharing platforms. The wide range of blockchain applications are ranging from certified data delivery services or certified notifications [22–24], to IoT platforms [25] like smart cities, supply chains, transport or health care as in the case of the present paper.

The implementation of the protocol presented in this paper uses the Ethereum blockchain, which uses the Ethereum virtual machine (EVM) to allow writing and executing code on the blockchain, programs known as smart contract. Concretely, is deployed over the Rinkeby test network, which one is a proof of authority network.

### Interplanetary file system

IPFS is a network designed to provide a method of storing and sharing content in a distributed peer to peer system, getting a censorship-resistant system [26]. One of the most important characteristics of IPFS is that it uses content addressing, in contrast to the well-known HTTP protocol that uses location addressing.

To obtain an efficient content addressing, IPFS identify every piece of content by a CID, that is the cryptographic hash of the content, then anyone who has the CID can access the data. The use of the CID for content addressing provides immutability since editing a file results to a new hash of that file, and duplicate files are only stored once since they always refer to the same hash [27].

Then, to link all data contained in the network (files and directories), IPFS uses Merkle Directed Acyclic Graphs (*Merkle* DAG) combined with a DHT (Distributed Hash Table) that performs the content rounting.^3^

IPFS nodes are not required to store all the data contained in the network, generally stores the data like in a cache [28], then, the garbage collector periodically will remove the content of the node. So, there is the possibility to remove entirely a data content of the network. For this reason exists the pinning service, which permanently attaches a certain content to a node, i.e. it is excluded from being deleted by the garbage collector [27].

Nowadays, the IPFS system is highly used in different secure blockchain protocols where it is needed an off-chain distributed storage system. In many cases it is used to reduce the high costs of blockchain storage [29, 30], store all the collected data by IoT systems [31] or as storage system for confidential medical registry [32–34].

In the implementation we have used the infura IPFS gateway, to perform the documents upload and download. The infura IPFS service also provides us the pinning of the documents until six months later the last access or the document upload.

### Proxy re-encryption

The proxy re-encryption (PRE) is a special type of public key encryption that provides the functionality to share encrypted data with third parties, without the need to decrypt the data and re-encrypt again with the cryptographic keys of the recipient. Concretely, it provides Alice (the encrypted data owner) a method to generate a re-encryption key from her private key and the public key of the recipient. Then, the re-encryption key, permits re-encrypt the encrypted data that will only be decryptable with the private key of the recipient. The fundamental point is that the proxy does not have to learn anything about the underlying plaintext or the secret keys of the involved actors [35].

The PRE concept was first introduced in 1998 by Blaze, Bleumer and Strauss in [36], where they propose the construction of the first implementation of a bidirectional proxy re-encryption with multiple uses. But it is not resistant to joint attacks between the proxy and a third party to which the access permission is attributed.

Essentially we have selected a threshold scheme because the security of a PRE with a single proxy is challenging (e.g. defending against collusion attacks problematic). So, the threshold scheme prevents third-party servers from converting encrypted data against the wishes of the file’s owners [37]. The re-encryption functionality is distributed between multiple proxies, achieving the decentralisation of the scheme, also provides a high security. The main goal of threshold PRE is to combine unidirectional PRE with a secret sharing to prevent access delegation of data against the wish of the data owner.

**Fig. 1:**
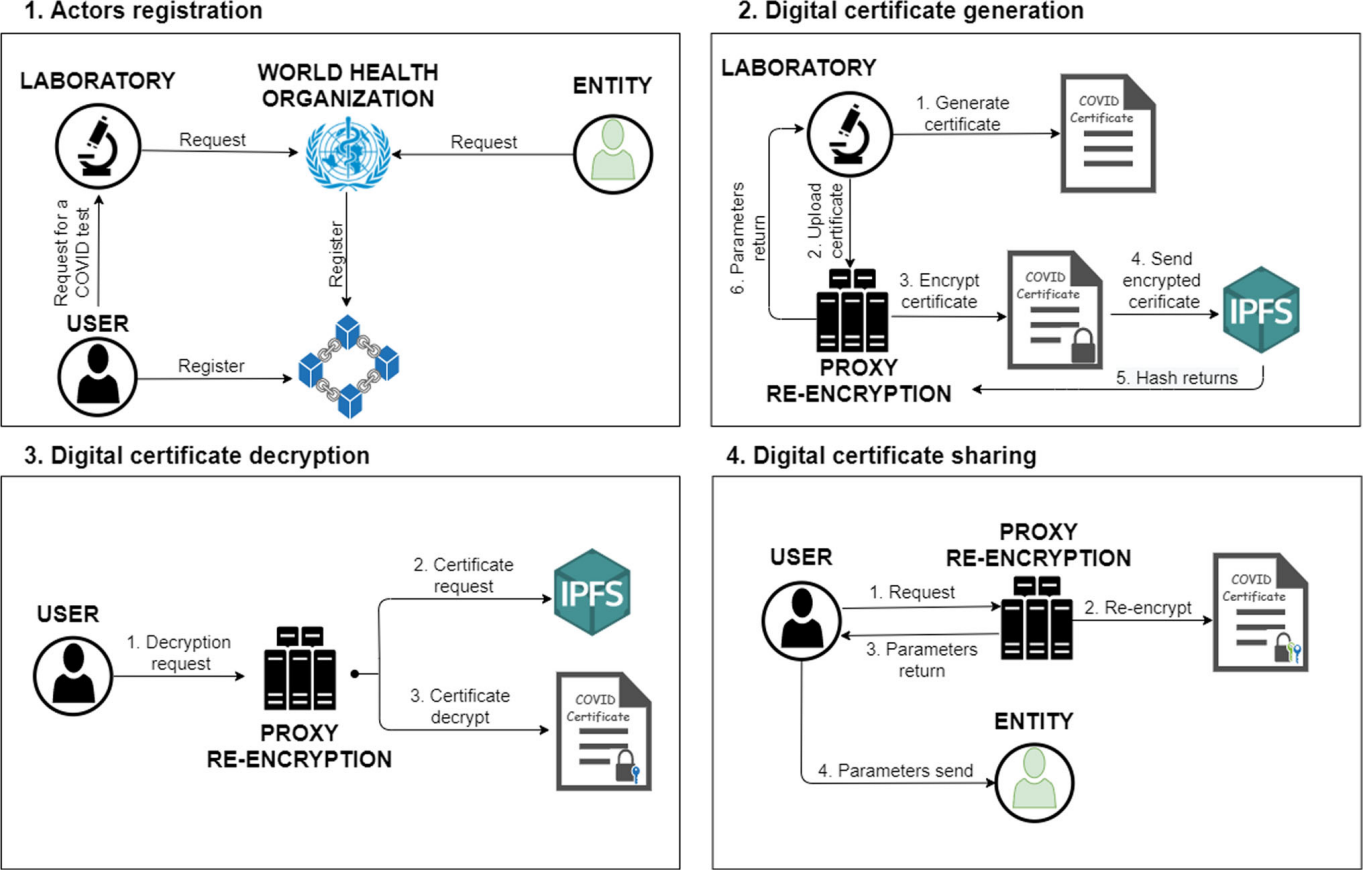
Main operation of the designed protocol diagram

To obtain this characteristics set, the threshold PRE scheme uses a set of *N* proxies and requires that at least *t* proxies re-encrypt the ciphertext before it is transformed to a valid re-encrypted ciphertext. So, with this idea it guarantees that if a certain number of proxies fail or get compromised, the scheme would not be vulnerable [38].

The use of PRE is extended for use in protocols that needs to achieve self-sovereignty in the share of confidential data, meaning that have to share the data in a highly securely and privacy form [39, 40].

## System overview

This section presents an overview of the system, describing the participating actors and the possible interactions among them. The followings are the principal actors that interact in the protocol:

- **Regulatory authority**. Entity in charge of validating laboratories to be part of the system, so that they can generate digital COVID-19 certificates for their patients. It is also in charge of the management of the trusted entities that are part of the system. In the implementation achieved it is represented by the World Health Organization (WHO).
- **Laboratory or testing centre.** Authority with the appropriate permission to generate new digital COVID-19 certificates for its patients introducing the COVID-19 tests results.
- **User.** Client of a laboratory requesting a digital COVID-19 certificate. It also has the functionality to share its certificates with requesting entities or system users.
- **Trusted entity.** Organisation that makes a request to the regulatory authority in order to be accredited as a trusted entity, i.e. an entity verified by the regulatory authority, which will be able to verify a large number of digital COVID-19 certificates. Some possible examples are: airlines, universities, companies in the tourism sector...
- **Non-trusted entity.** Small organisation that want to provide secure services to their clients, but they perform the verification phase like a user actor which are determined by the regulatory authority intervention and can only verify a limited number of digital COVID-19 certificates. Some possible examples can be: music schools, sport clubs, small restaurants...

**Fig. 2:**
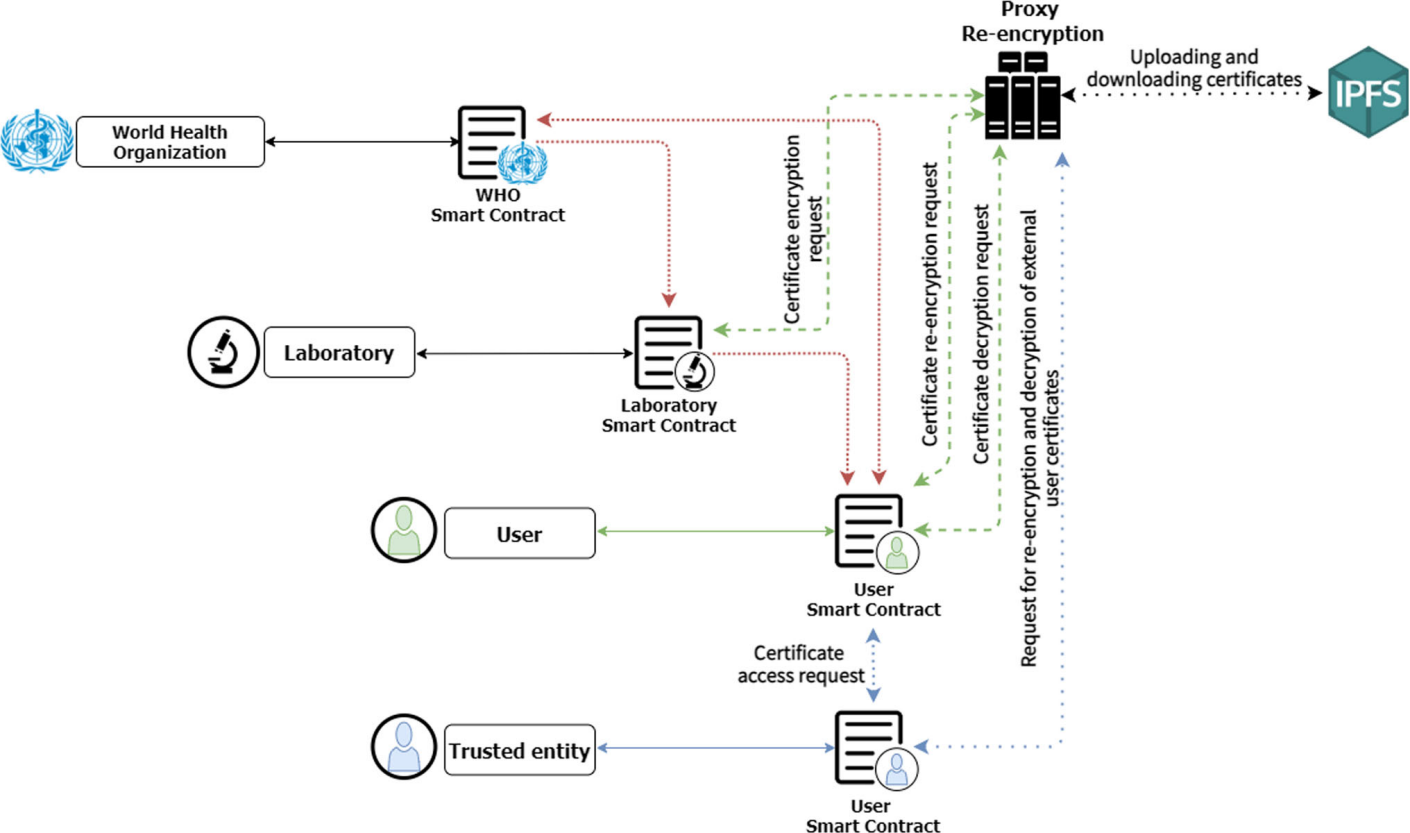
Diagram of the main communication of the protocol with the smart contracts implemented

The main operation of the designed protocol is represented in Fig. 1 and is described in the following steps:

1. The different actors must register to the system in order to obtain all its functionalities. The laboratories and entities registration functionality are executed by the WHO, because it has to verify that the requester complies all the requirements.
2. The users, externally to the system, request a registered laboratory to perform a COVID-19 test. The laboratory will generate a digital COVID-19 certificate with the results of the test. It will be encrypted by the PRE service and then stored in the IPFS storage system. Finally, the laboratory delivers all the parameters of the encrypted certificate to the user owner.
3. The owner users can obtain their digital COVID-19 certificate at any time requesting to the PRE service the download from the IPFS and the decryption of it.
4. The owner users also can share their digital COVID-19 certificate with different trusted entities or other system users for their digital COVID-19 certificate verification, requesting re-encryption to the PRE service.

As indicated above, in addition to trusted entities, users of the system (and non-trusted entities) can verify external certificates. The protocol proposes this functionality because it is considered possible that small organisations want to be able to offer secure services to their customers. Therefore, without the need to make a trusted entity validation request, any user can request and verify external certificates, but such requests will always be controlled by the regulatory authority. The users can never share a digital COVID-19 certificate directly to another user or non-trusted entity of the system; it must always be verified by the regulatory authority.

Particularly in the implementation of the protocol, all the participating actors possess blockchain addresses and are able to communicate with the three different smart contracts defined. Figure 2 shows the three defined smart contracts, the communications between them and the PRE service defined in the protocol.

The solid lines of Fig. 2 represent the relationship between the actor owner of the smart contract and its smart contract, the red dashed lines represent different communications between smart contracts whether they can be bidirectional or unidirectional. Particularly, the WHO smart contract can send information to the different laboratory smart contracts and also to the user smart contract. The laboratory smart contracts can only send information to the user smart contracts and the user smart contracts to the WHO smart contract, as it will be explained in detail in Sects. 6 and 7.

The black dashed line represents the interaction between the PRE service and the IPFS distributed storage system, where the digital COVID-19 certificates are stored, this communication cover the upload of the encrypted digital COVID-19 certificates and the downloads for future decryptions.

The green dashed lines represent the communication between the laboratory and the PRE service to perform the encryption request. And the communication between the user who owns the digital COVID-19 certificate and the PRE service to perform the re-encryption of the certificate and allow access to it by another user of the system.

At last, the blue lines represent the request by a trusted entity for access to an external digital COVID-19 certificate for verification, as well as the interaction with the PRE service for the completion of the requested certificate re-encryption key generation and the corresponding decryption.

## Protocol

The protocol designed for the creation and management of digital COVID-19 certificates is based on seven distinct phases. The first three phases involve the registration of the different actors (laboratory management, user registration and trusted entities management), followed by two phases for the generation of new digital COVID-19 certificates by the accredited laboratories (introduction of digital COVID-19 certificates by laboratories and decryption of digital COVID-19 certificates by the owner) and finally two more phases for sharing the digital COVID-19 certificates with trusted entities or other users of the system (digital COVID-19 certificate sharing and decryption of external certificates).

### Actors registration

#### Laboratory management

The first phase of the protocol is laboratory management, a task carried out by the regulatory authority (the WHO) that defines the flow of steps for the addition of new laboratories and the corresponding removal of laboratories from the system.

**Admission of laboratories**

**Fig. 3:**
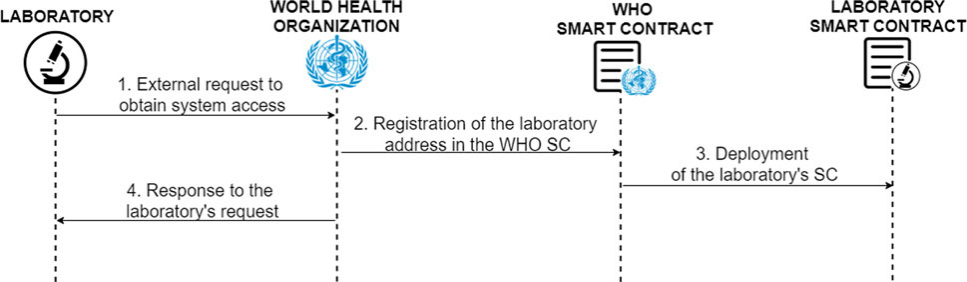
Diagram of the protocol for the admission of new laboratories

In case a laboratory wants to be able to generate COVID-19 accredited certificates, first it must make a request to the WHO for admission to the system as a certifying laboratory (1), as it can be seen in Fig. 3. Once the WHO has validated the laboratory, it is added to the system (2), using the Ethereum address provided by the laboratory.

**Fig. 4:**
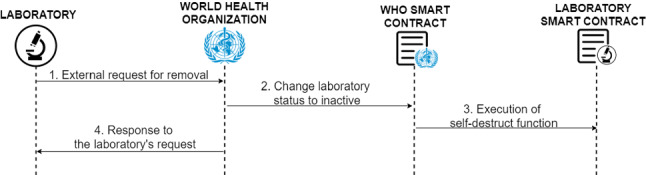
Diagram of the protocol for the removal of laboratories

The WHO enters the laboratory’s address and the representative name of the laboratory in the system, then the laboratory is stored in the list of laboratories, and the WHO smart contract deploys the smart contract representative of the laboratory that has been registered (3).

Finally, the WHO sends an external notification to the requesting laboratory indicating whether it has been possible to introduce the laboratory into the system or, if not, the reason(s) why the laboratory has not obtained the accreditation (4).

If the laboratory has obtained accreditation, as soon as the WHO notifies the resolution to the laboratory, it can provide the digital COVID-19 certificate generation service to its users.

**Laboratories discharge**

To completely remove the permissions of a laboratory, the WHO has the power from its smart contract to completely destroy the smart contract representing the laboratory from the blockchain and change the status with which it is stored within the WHO smart contract, the removal status is activated.

This functionality is represented in Fig. 4, it can only be executed if the laboratory has made the corresponding request (1), or if a dishonest behaviour, which could have an effect on global health, of the laboratory is detected. When it is received or detected the WHO change the laboratory status to inactive (2) and executes the self-destruct function (3) of the laboratory smart contract. Functionality executed when the WHO enters the Ethereum address of the laboratory to whom it wants to remove the accreditation. Finally, the WHO needs to inform the laboratory through a notification using an external medium (4).

#### User registration

As a first step of the user registration phase, users have to obtain their public and private key pair, so that the PRE service can carry out the corresponding encryption of the digital COVID-19 certificates.

**Fig. 5:**
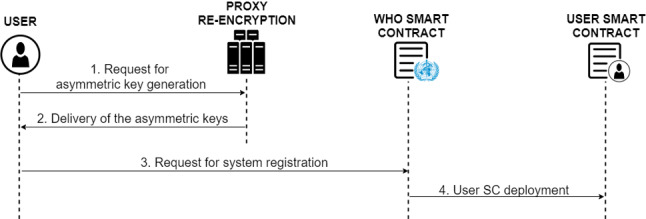
Diagram of the protocol for user registration

Therefore, the first action of the registration phase, as it can be seen in Fig. 5 is the request from the users to the PRE service to generate the encryption key pair (1, 2). Then, users will share their public key with the rest of users of the system, and the private key of each user must be kept confidential.

Once the user has their key pair, the registration request is made to the system (3), where the requesting user with their Ethereum address makes a request to the WHO smart contract. Consequently, a new smart contract owned by the user is deployed (4), containing all its functionalities. In addition, the Ethereum addresses of the user and the smart contract are registered in the WHO smart contract.

As the protocol is defined, it is not necessary for a user to be a patient of a single laboratory, a user can obtain digital COVID-19 certificates from multiple laboratories.

#### Trusted entities management

The trusted entities represent all organisations/entities that request access to the system in order to be able to verify a large number of digital COVID-19 certificates of the system users. Once the trusted entity attribute has been obtained two main benefits are achieved:

- Direct sending of a digital COVID-19 certificate between a user and the trusted entity, without the intervention of the WHO control verifying the identity of the entity.
- Sending of requests directly to the users without the WHO verification filtering.

**Fig. 6:**
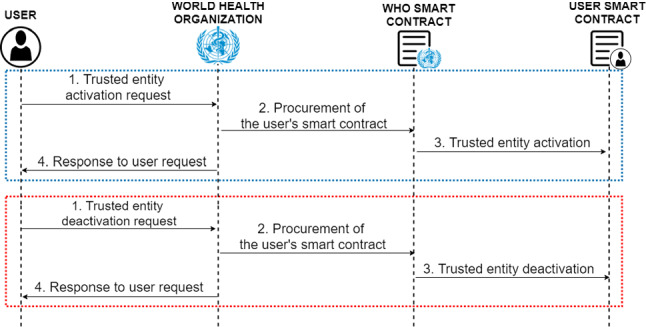
Diagram of the protocol for trusted entities management

In order to achieve the activation of the trusted entity attribute, previously the entity or organisation has to be registered as a system user. Then the following set of steps represented in the blue box of Fig. 6 must be performed.

First, it is necessary for the entity to make the corresponding request to the WHO, through an external channel to the system (1). The WHO needs to verify all the information received from the requesting entity to ensure that the entity is who it claims to be and that it meets all the requirements imposed by the regulatory authority in order to be accredited as a trusted entity. In case the verification is positive, the WHO proceeds to activate the entity’s smart contract as a trusted entity (2, 3). Finally, the WHO replies to the entity’s request by indicating that the verification has been positive and the activation of the trusted entity attribute has been carried out (4). Otherwise, the WHO replies to the entity indicating that the verification has not been positive (4).

In the case of deactivation of the trusted entity feature, shown in the red box of Fig. 6, two cases are distinguished. First, the case in which the entity makes a request for such deactivation, and the case in which the WHO considers that the user is untrustworthy, then the WHO can immediately execute the deactivation.

When the entity wants deactivation, it must first make the corresponding request (1). The WHO then verifies that the applicant is who it claims to be. If the verification is positive, the WHO proceeds with the deactivation (2, 3). Otherwise, for example, if impersonation is detected, the request is denied. Finally, the WHO notifies the owner of the entity about the resolution of the request (4).

In any case, it is important to note that once the deactivation of such a feature has been carried out, the WHO must notify the owner of the entity indicating the corresponding causes.

### New digital COVID-19 certificates generation

#### Introduction of digital COVID-19 certificates by laboratories

The procedure between users and laboratories to carry out the necessary test to issue the digital COVID-19 certificates are outside the scope of the proposed system in this paper.

**Fig. 7:**
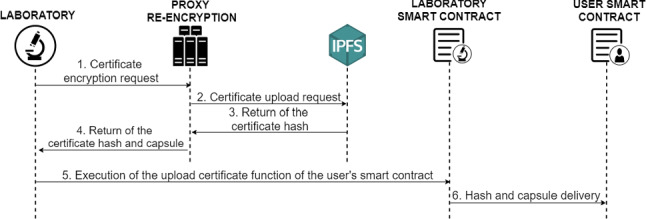
Diagram of the protocol for introduction of digital COVID-19 certificates

Then, once the results of the tests have been obtained, the laboratory can introduce the users digital COVID-19 certificates, but first it is necessary that the users have provided their Ethereum addresses in order to link the new document with the owner users. The digital COVID-19 certificates particularly contain the patient information, the PCR test, antibody test or vaccination information and also the validity period of the certificate.

Figure 7 shows that before uploading the document, the laboratory makes a request to the PRE service (1), which is responsible for encrypting the document using the public key of the owner user. It is also in charge of uploading the encrypted document to the IPFS distributed storage system (2), which returns the hash (document identifier) of the digital COVID-19 certificate uploaded to the PRE service (3).

Once the document has been uploaded, the PRE service must return the hash and capsule to the laboratory (4), responsible to send this information to the owner’s smart contract of the encrypted digital COVID-19 certificate (5), who is the only one capable of the decryption of the certificate.

The proxy re-encryption scheme used in the protocol employs a KEM/DEM approach [41], instantiated over elliptic curve secp256k1, in which an ephemeral symmetric key is used to encrypt the data, and the symmetric key is encrypted using an asymmetric encryption key. The encrypted data (*encData*) and the encrypted symmetric key (*capsule*) are stored together [42].

For the encryption of the symmetric key it should be noted that the KEM mechanism does not encrypt the symmetric key, it encrypts a random value of the size of the asymmetric key (in order to avoid padding) from which the symmetric key is derived

First of all, for the encryption of the digital COVID-19 certificate, the PRE service in the KEM/DEM construction employs the encrypt algorithm, using the owner user public key ($pk_A$) provided by the laboratory and the digital COVID-19 certificate as message (*M*).

1 $$\begin{aligned} \text {Encrypt}(pk_A, M) = C \end{aligned}$$

Then, the encrypt algorithm performs the following steps:

1. The KEM construction using $pk_A$ encapsulate a symmetric key *K* and a *capsule* that allows deriving again the symmetric key *K*.
  2 $$\begin{aligned} (K, capsule) = \text {Encapsulate}(pk_A) \end{aligned}$$
2. The encrypted message (*encData*) is obtained applying AEAD [43] with input parameters *K* and *capsule* over *M*:
  3 $$\begin{aligned} encData = encrypt_\mathrm{AEAD}(K, capsule)\left\{ M\right\} \end{aligned}$$
3. Finally, *C*, the ciphertext of the digital COVID-19 certificate consists of:
  4 $$\begin{aligned} C = (capsule, encData) \end{aligned}$$

#### Decryption of digital COVID-19 certificates by the owner

**Fig. 8:**
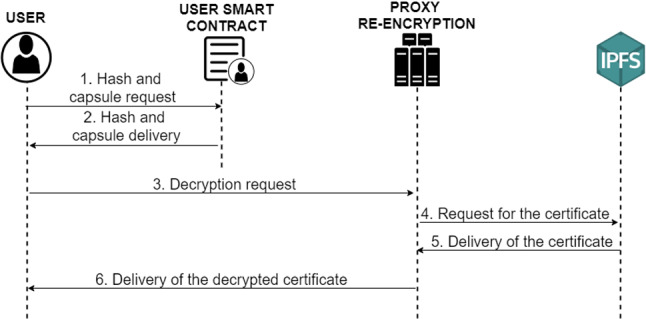
Diagram of the protocol for decryption of owned digital COVID-19 certificates

To decrypt a digital COVID-19 certificate owned by the user, it is first necessary for the user to obtain the hash that identifies the certificate within the IPFS system and the encryption key of the certificate, information that can be found within the user’s smart contract (1, 2). Therefore, a request is made to obtain these parameters, like it is shown in Fig. 8.

Once these necessary parameters have been obtained, a request is made to the PRE service to decrypt the digital COVID-19 certificate (3). Prior to the decryption phase, the PRE service must obtain the certificate requested by the user, i.e. it must make the corresponding request to the IPFS service indicating the hash of the document to be obtained (4, 5). The PRE service, using all the parameters obtained by the user and the digital COVID-19 certificate requested to IPFS, proceeds to decrypt the document. Finally, if no error has occurred, the decrypted certificate is returned to the user (6).

The decryption algorithm used by the PRE service [42] is defined as follows, applying the secret key ($sk_A$) provided by the user owner, and the digital COVID-19 certificate ciphertext (*C*, formed by *encData* and *capsule*), the KEM/DEM algorithm performs:

5 $$\begin{aligned} \text {Decrypt}(sk_A, C) = M \end{aligned}$$

The decrypt algorithm performs the following steps:

1. The KEM algorithm to obtain the decrypted digital COVID-19 certificate decapsulate the symmetric key *K* using the user secret key ($sk_A$) and the corresponding *capsule*, the algorithm outputs the symmetric key *K*, or $\perp$ if the symmetric key is invalid.
  6 $$\begin{aligned} K = \text {Decapsulate}(sk_A, capsule) \end{aligned}$$
2. Decrypts the *encData* ciphertext using the AEAD decrypt algorithm [43] obtaining the plaintext of the digital COVID-19 certificate (*M*), or $\perp$ if decryption fails.
  7 $$\begin{aligned} M = \text {decrypt}_\mathrm{AEAD}(K, capsule)\left\{ encData\right\} \end{aligned}$$

### Digital COVID-19 certificates sharing

#### Digital COVID-19 certificate sharing

For the sharing of digital COVID-19 certificates, the protocol distinguishes different procedures depending on whether the recipient is a trusted entity or a user (non-trusted entity):

**Fig. 9:**
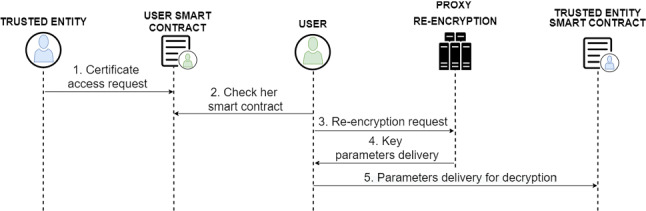
Diagram of the protocol for digital COVID-19 certificate requests by trusted entities

**Fig. 10:**
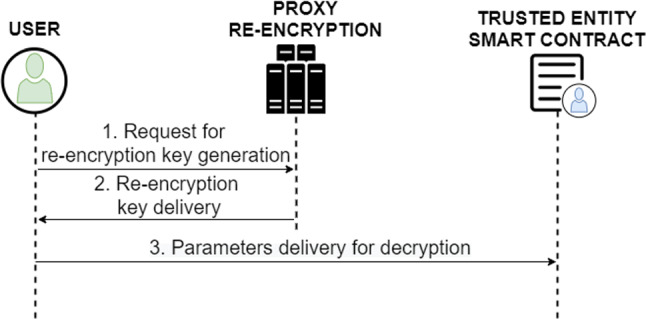
Diagram of the protocol for digital COVID-19 certificate send to trusted entity

**Fig. 11:**
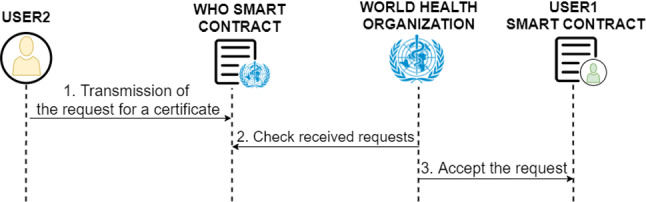
Diagram of the protocol for digital COVID-19 certificate send to another system user

1. **Trusted entity**
  **Request for the certificate by a trusted entity.** In order to be able to make a request for a digital COVID-19 certificate from a trusted entity, it is first necessary for the entity to know the Ethereum address of the users to whom it wants to make the request. Then, once it has the Ethereum address at its disposal, it can make the request directly to the smart contract of the users who owns the certificate (1), thanks to the fact that it has the trusted entity feature activated, like it can be shown in Fig. 9. The owner users must then check that they know the requesting Ethereum address and decide whether they want to share access to one of their digital COVID-19 certificates or not (2). If the owner accepts the request, a request is made to the PRE service to re-encrypt the digital COVID-19 certificate encryption key (3), which the PRE service will respond with all the necessary parameters to be able to perform the correct decryption (4). Finally, all the necessary information, such as the hash (identifier) of the certificate in the IPFS system and the parameters of the re-encryption key, is sent to the smart contract of the requesting entity (5), so that it has all the necessary information to be able to decrypt the digital COVID-19 certificate.
  **Direct sending of the digital COVID-19 certificate.** In the case where the owner user wishes to share the digital COVID-19 certificate directly without a prior request to give access to a trusted entity, the user must know the Ethereum address of the entity, then enter it together with the hash of the certificate. The same sharing process is then carried out as explained in the previous section and shown in Fig. 10.
2. **User** In order to send a digital COVID-19 certificate from one user of the system to another user (non-trusted entity), it is necessary that the user receiving the certificate (user2) first makes a request to the user owning the certificate, as shown in Fig. 11. This request will first be sent to the WHO smart contract (1), and the WHO will carry out an initial check to verify that the recipient is not a user with bad reputation or intentions (2). Therefore, if the WHO considers the request to be valid, it is sent to the recipient user (3). Otherwise, if the WHO detects that the requesting user is not trustworthy, it will consider the request as invalid. In the case that the WHO accepts the request, the owner user has two options:
  If the owner user knows the Ethereum address, can accept it and then enter the hash of the digital COVID-19 certificate to proceed with the re-encryption of the encryption key, as is done in the previous shares.
  If the user does not know the Ethereum address or does not want to share any digital COVID-19 certificate with this Ethereum address, can consider the request as resolved without performing the re-encryption.

The encryption algorithm used in the digital COVID-19 certificate re-encryption cases used by the PRE service [42] is named ReEncrypt and outputs the re-encrypted ciphertext $C'$.

8 $$\begin{aligned} \text {ReEncrypt}(kFrag, C) = C' \end{aligned}$$

Then, the ReEncrypt algorithm performs:

1. The execution of the KEM ReKeyGen algorithm for the re-encryption key fragments generation. On input the secret key of the user that shares the digital COVID-19 certificate ($sk_A$), the public key of the receiver ($pk_B$), a number of fragments (*N*) and a threshold (*t*). Then, it computes *N* fragments of the re-encryption key between the two users, named *kFrag*.
  9 $$\begin{aligned} kFrag = \text {ReKeyGen}(sk_A, pk_B, N, t) \end{aligned}$$
2. The performance of the capsule fragment (*cFrag*) generation, with the execution of the ReEncapsulation algorithm. Where on input a *kFrag* and a *capsule*, outputs the *cFrag*, or $\perp$ if the decryption fails.
  10 $$\begin{aligned} cFrag = \text {ReEncapsulation}(kFrag, capsule) \end{aligned}$$
3. Finally, the re-encrypted ciphertext of the digital COVID-19 certificate is defined by:
  11 $$\begin{aligned} C' = (cFrag, encData) \end{aligned}$$

#### Decryption of external digital COVID-19 certificates

For the decryption of external digital COVID-19 certificates, only the communication between the receiving user and the proxy re-encryption service is necessary, as well as the execution of queries between the user and his smart contract to obtain all the necessary parameters.

**Fig. 12:**
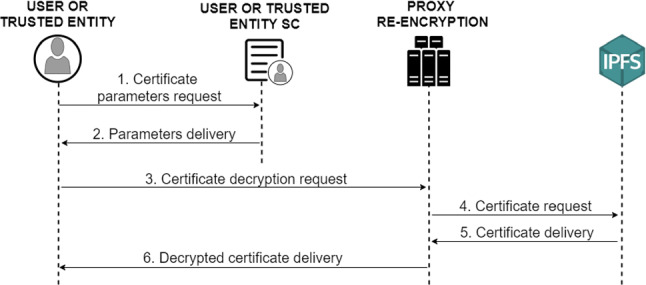
Diagram of the protocol for decryption of external certificates

Specifically, the following procedure is followed, the user or trusted entity performs the query in its smart contract to obtain all the parameters that the user who owns the document has entered in the previous phase (Fig. 12 steps 1, 2). It then sends these parameters to the PRE service (3), which downloads the digital COVID-19 certificate stored in the IPFS system (4, 5) and decrypts it with the re-encryption key generated. Finally, the PRE service transmits the decrypted digital COVID-19 certificate to the requesting user (6).

The decryption algorithm used in the re-encryption digital COVID-19 certificate cases used by the PRE service [42] is named DecryptFrags and outputs the plaintext (*M*) of the digital COVID-19 certificate or $\perp$ if decryption is invalid. On input it employs the secret key ($sk_B$) of the smart contract receiver user and a set of *t* re-encrypted ciphertexts ($C'$) formed by a *cFrag* and the *encData*.

12 $$\begin{aligned} \text {DecryptFrags}(sk_B, \left\{ C'_i \right\} ^t_{i=1}) = M \end{aligned}$$

1. The KEM approach, decapsulates the *cFrags* and produce the key *K*, or $\perp$ if decryption fails:
  13 $$\begin{aligned} \text {DecapsulateFrags}(sk_B, \left\{ cFrag_t\right\} ^t_{i = 1}) = K \end{aligned}$$
2. The DEM approach, decrypts the ciphertext *encData* using the decrypt AEAD algorithm [43], obtaining the plaintext of the digital COVID-19 re-encrypted certificate (*M*), or $\perp$ if decryption fails:
  14 $$\begin{aligned} M = \text {decrypt}_\mathrm{AEAD}(K, capsule)\left\{ encData\right\} \end{aligned}$$

## Smart contracts

The implementation of smart contracts realised from the designed protocol as already introduced with Fig. 2 is constituted by the definition of three smart contracts linked among them, which perform the necessary communications to achieve the purposes of each one, following the definition of the protocol. Specifically, the WHO or regulatory authority, the laboratories and the end users of the system are developed as smart contracts. Next, the main functions of each smart contract that enable the correct performance of the protocol are introduced.

### Regulatory authority smart contract

The regulatory authority smart contract defines all functions that allow the laboratories, users and trusted entities management. It allows also the management of digital COVID-19 certificates requests between system users.

First, before introducing the explanation of the main functions of the regulatory authority’s smart contract, it should be noted, in Listing 1, that the owner’s Ethereum address is defined as a variable (*owner*), i.e. in the case of the WHO representing the regulatory authority, the address owned by the WHO is entered. In such a way that it is easy to detect which address is the owner.

#### Laboratories management functions

Two functions are defined for the laboratories management, registerLab() and deleteLab(), which as the name suggests allow the introduction and removal of laboratories to the system.

First, in the registerLab() function, Listing 2, a new laboratory smart contract is deployed from the laboratory owner Ethereum address. In addition, the information relating to the laboratory that is being added to the system is stored in the regulatory authority’s smart contract, using the *struct_Lab* structure.

Also when this function is executed the newLab() event is triggered to publish that a laboratory has been added in the system and to indicate the name of the laboratory and the corresponding Ethereum address.
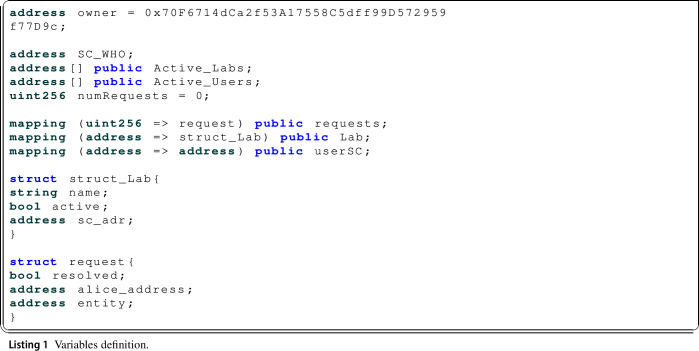

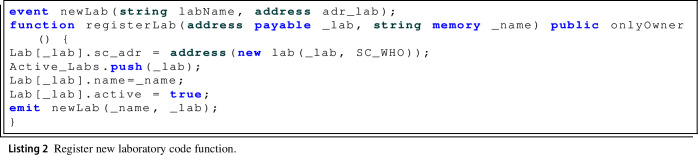


The deleteLab() function, Listing 3, only changes the value of the Boolean parameter of the *struct_lab* structure, which is intended to allow control over whether a laboratory is active or inactive in the system. In addition, to disable the laboratory from further use of the system from this function it calls the destruct() function of the smart contract of the laboratory where the function selfdestruct() of the smart contract is executed. Also when this function is executed the labDeleted() event is triggered to publish that a laboratory has been removed from the system and to indicate which laboratory it is. 
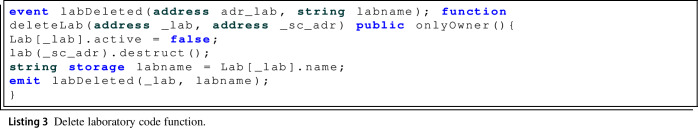


#### Users management functions

As far as the registration of new users of the system, the main function is the one called registerUser(), Listing 4, which allows new users to be registered. In this function, the user’s smart contract is deployed with the definition of the *sc_addr* variable.

As can be seen, for the deployment of the user’s smart contract, it introduced the owner address of the smart contract, the public key generated by the PRE service and the address of the WHO smart contract, necessary parameters for the execution of the user’s functions. Also, the user’s address and the address of the smart contract that has just been deployed are stored in the WHO smart contract in order to be able to carry out the different queries and transactions. Finally, when this function is executed the new_User() event is triggered to publish that a user has been added to the system and to indicate the user Ethereum address. 
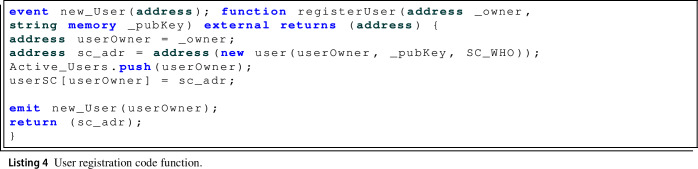
 The rest of the important user management functions refer to the resolution of requests to send digital COVID-19 certificates between users or between a user and a trusted entity, requests which, as already introduced, are first sent to the WHO smart contract.

First of all, it is the getAliceDocs() function, Listing 5, used by the different registered users of the system and trusted entities that request a digital COVID-19 certificate from another registered user. 
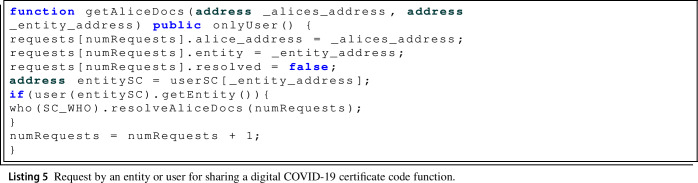
 In addition, as the protocol indicates, it is necessary to check if the requesting address corresponds to an active trusted entity, as in this case the request is directly resolved by the WHO and sent directly to the recipient user, by executing the resolveAliceDocs() function, Listing 6. The getAliceDocs() function as it is expected could only be executed by a system registered user, so the onlyUser() modifier is applied. 
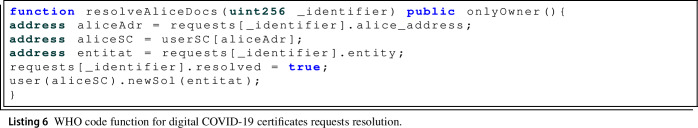
 The resolveAliceDocs() function is used in two cases, when the WHO wants to positively resolve a request by sending a digital COVID-19 certificate between two users, and in the case where the issuer of the request is a trusted entity, case where the WHO allows the request to be resolved directly.

Therefore, in this function, through the *identifier* parameter, all the information corresponding to the identified request is obtained, and it is sent to the smart contract of the recipient user. In addition, the request is considered positively resolved from the WHO’s point of view.

Finally, of the user management functions, the deny-Sol() function, Listing 7, is used to consider a request resolved, but in this case it is not forwarded to the recipient user, i.e. it represents the case in which the WHO denies the request received between two users (or between a user and a non-trusted entity). 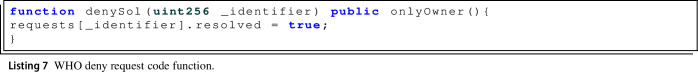


#### Trusted entities management function

For the management of trusted entities, the entity() function is defined, Listing 8, where indicating the owner address of the user’s smart contract (_entityAdr), the WHO can activate or remove the trusted entity attribute of the specified smart contract, calling the user activeEntity() function, where the user entity variable simply takes the Boolean value entered as a parameter. Also when this function is executed the Entity() event is triggered to publish that the entity attribute has changed the state.

Finally, emphasise that some functions defined in the WHO smart contract uses the *onlyOwner()* modifier, which one restrict that this functions only can be executed from the smart contract owner address or from the WHO smart contract address. The rest of functions, being public, can be executed by any user. 
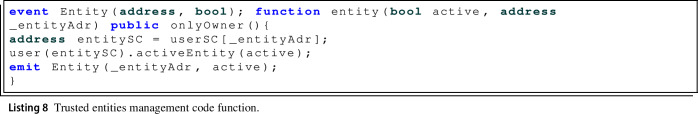


### Laboratory smart contract

The smart contract of laboratories is responsible for allowing the introduction of new digital COVID-19 certificates.

First, the *owner* variable is defined to store the Ethereum address of the laboratory owner and *whoSC_ Addr* to store the address of the regulatory authority, in order to impose that only this authority can execute the destruction function of the smart contract. 
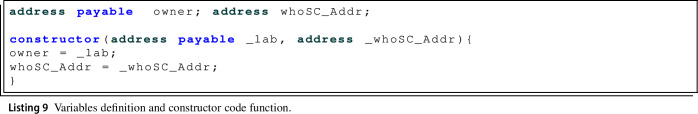
 Next, the constructor of the smart contract is defined, in which the storage of the indicated variables is simply carried out. Parameters that are introduced by the WHO.

The uploadCert() function represents the delivery of the digital COVID-19 certificate to the owner user. It is based on the introduction of the Ethereum address of the digital COVID-19 certificate owner to which the new certificate must be delivered, the IPFS hash to obtain the digital COVID-19 certificate in future occasions, and the capsule of the symmetric encryption key of the certificate generated by the PRE service, parameters delivered to the smart contract of the owner user. 
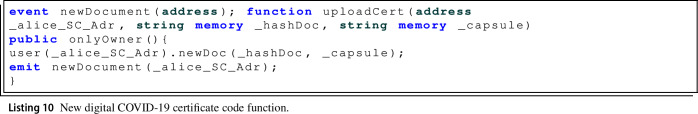
 In addition, the newDocument() event is emitted, from which it is published that a new digital COVID-19 certificate has been introduced into the system, indicating the Ethereum address of the certificate owner. It should be noted that no information about the content of the digital COVID-19 certificate is published, since thanks to the encryption carried out, based on the IPFS hash and the symmetric encryption key, the only user who can access the decrypted information is the owner.

As it can be seen in Listing 10, the uploadCert() function uses the onlyOwner() modifier, which restrict the upload of new documents from this smart contract to the owner address.

Finally, the smart contract of the laboratory also contains the function for the definitive removal of the laboratory from the system, the destruct() function, in which the destruction of the laboratory is carried out with Solidity selfdestruct() function. The destruct() function only can be executed by the regulatory authority, the WHO, so for that is used the onlyWHO() modifier. 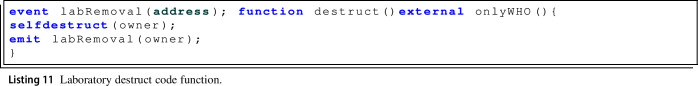


### User smart contract

The last smart contract defined is the users smart contract, which, as mentioned above, contains the trusted entity feature, i.e. it allows the functions of both entities and users to be carried out, depending on whether this feature is active or not. 
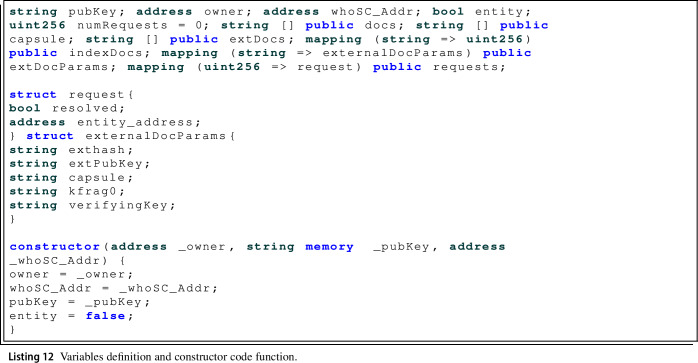
 First, as in the rest of the smart contracts, the set of variables to be used are defined, where it should be noted that not only the variables necessary to obtain the correct performance of the designed protocol are introduced, but also a set of variables whose functionality is focused on allowing an easy implementation of the user interface. The set of variables defined in this smart contract can be seen in Listing 12, jointly with the smart contract constructor.

It is important to take in mind the set of mappings defined, the first one *indexDocs* has the functionality of allowing from the hash of a digital COVID-19 certificate obtain its index in the array *docs* and *capsule*. The *extDocsParams* mapping also from the hash of an external digital COVID-19 certificate returns all the parameters of the certificate. And finally *requests*, from the request number returns the information of the request, defined in the *request* structure where a Boolean value is stored indicating whether the request has been resolved or not and the address of the entity or external user that is making the request (*entity_address*). Finally, the *externalDocParams* structure is defined where all the necessary parameters are stored in order to request the PRE service to decrypt a certificate of an external user that has shared access.

In order to be able to deploy this smart contract, the constructor is defined, which will be executed from the WHO smart contract call. The constructor stores the Ethereum address of the smart contract owner, its public encryption key and the address of the WHO smart contract, as well as initialising the trusted entity feature as inactive.

#### Digital COVID-19 certificates management functions

For the introduction of digital COVID-19 certificates owned by the smart contract, it is only necessary to store the IPFS hash where the encrypted certificate is located and the capsule of the symmetric encryption key generated by the PRE service. Therefore, in the newDoc() function, the hash and the capsule are introduced into the corresponding arrays, for on future occasions the certificate can be consulted. This function only can be executed by registered laboratories, as it is restricted by the onlyLab() modifier. 
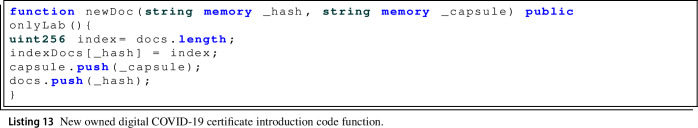
 On the other hand, the function for storing external certificates newExtDoc() requires a larger number of parameters, as it has to store all parameters to perform the re-encryption of the symmetric encryption key. Specifically, an array is used to store the set of identifiers (hash) of the digital COVID-19 certificates stored in IPFS and the *externalDocParams* structure where the information to be sent to the PRE service with the user’s encryption keys is stored in order to decrypt the chosen certificate. The newExtDoc() function is used by users that want to share their digital COVID-19 certificate with the smart contract owner, so this function only can be executed by a registered user, as it is restricted by the onlyUser() modifier. 
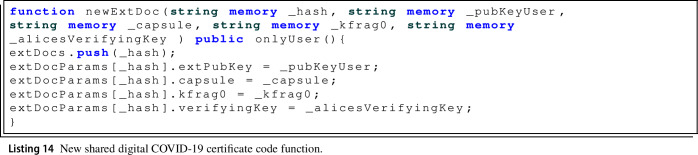


#### External requests management functions

In the management of external requests, the newSol() function is defined, which is executed when a request received from a trusted entity or user is sent from the smart contract of the regulatory authority, i.e. when the regulatory authority considers a request to be resolved and finally sends it to the recipient user. In order to have control of the different requests received in the newSol() function, the address of the trusted entity or user that sends the request is stored in the request structure. To determine that only the WHO smart contract can execute this function, is defined and used the onlyWHO() modifier. 
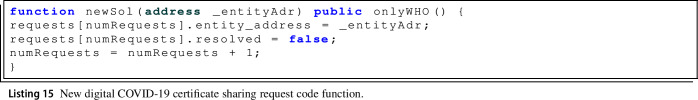


Next, the resolveSol() function is defined, where by introducing the request identifier this is marked as resolved. The request identifier corresponds to the request number, a value that is initially zero and increases by one value for each new request received. As expected, this function can only be executed by the smart contract owner, so the onlyOwner() modifier is defined. 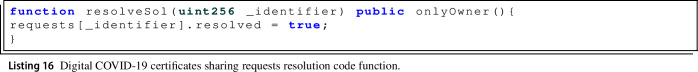


## Security and privacy analysis

This section presents a security analysis of the system. The analysis presents two parts. In the first one the most important properties that the protocol provides are discussed. In the second part, we present the results of a vulnerability analysis of the code.

### Properties

The proposed system fulfils the properties of availability, integrity, confidentiality, authentication, non-repudiation, self-sovereignty, immutability, auditability, authorisation and traceability. Below, each one of these properties is evaluated.

**1.**: **Availability**

#### Proposition 1

Digital COVID-19 certificates are available to authorised parties.

#### Claim 1

The encrypted certificates can be always and universally available.

#### Proof

The digital COVID-19 certificates are stored, encrypted, in the IPFS system, where they can be always accessible to everybody. To obtain permanent accessibility, the system uses the infura pining service, which provide the permanent storage of content, up to six months after the last access. Also, it is possible to obtain permanent access without restrictions using a proprietary IPFS node, where the digital COVID-19 certificates would be stored. $\square$

#### Claim 2

It is possible provide permanent access to the user who owns a digital COVID-19 certificate to the information contained in the certificate.

#### Proof

The certificate information is stored encrypted in the IPFS and the information for its decryption is in the smart contract of the user, so the user is capable to decrypt their certificates at any moment.$\square$

#### Claim 3

It is possible to provide permanent access to authorised external users/entities to the information contained in the digital COVID-19 certificate.

#### Proof

The digital COVID-19 certificate information is stored encrypted in the IPFS system and the information for its decryption will be sent to the PRE service to decrypt the certificate. External authorised users have access to all the decryption parameters to obtain the digital COVID-19 certificate. $\square$

**2.**: **Integrity**

#### Proposition 2

Through the use of the PRE service the proposed protocol provides data integrity.

#### Claim 4

The integrity of the digital COVID-19 certificates encrypted are guaranteed.

#### Proof

Blockchain is a technology that by itself provides the integrity feature. The encrypted digital COVID-19 certificates are stored in the IPFS, and its hash is used in the protocol. This way the encrypted digital COVID-19 certificate cannot be modified. $\square$

#### Claim 5

The integrity of the information contained in the digital COVID-19 certificates are guaranteed.

#### Proof

If any parameter that makes up the encryption of a digital COVID-19 certificate (symmetric encryption key, public and private keys of authorised users, certificate hash...) or the certificate itself is modified, the PRE service will no longer be able to decrypt the digital COVID-19 certificate, thus detecting that any parameter has been modified. $\square$

**3.**: **Confidentiality**

#### Proposition 3

The digital COVID-19 certificates are confidential.

#### Claim 6

The digital COVID-19 certificates are stored publicly in the IPFS, but they are encrypted and private.

#### Proof

The digital COVID-19 certificates are confidential. Although they are stored in a public file system, as IPFS, its contents are not accessible to the general public. $\square$

#### Claim 7

Only authorised users can access the digital COVID-19 certificates information.

#### Proof

The digital COVID-19 certificates are confidential thanks to the use of the PRE service and asymmetric cryptography. The parameters for the decryption of the certificates are stored publicly in the user’s smart contract, but without the use of the private key of the user with access to it, it is not possible to decrypt the digital COVID-19 certificate, and as it has already been indicated above, this private key must be kept by the user in the most secure way. $\square$

**4.**: **Authentication**

#### Proposition 4

The proposed protocol provides data authentication.

#### Claim 8

The regulatory authority keeps all the information regarding the users and their corresponding smart contracts.

#### Proof

The protocol provides data authentication thanks to the fact that the regulatory authority, in its smart contract, contains all the information regarding the Ethereum addresses of the system users and their corresponding smart contracts. In addition, in order to obtain the title of trusted entity, the regulatory authority must request information about the fulfilment of the necessary requirements and it will also verify that the entity is not an impersonation. $\square$

#### Claim 9

It is always possible to identify who is the issuer of a transaction.

#### Proof

Thanks to the use of blockchain technology, the information of the Ethereum address issuing the transaction is stored for all the transactions; therefore, it is always possible to know who is the issuer of the transaction. $\square$

**5.**: **Non-repudiation**

#### Proposition 5

The system provides the service of non-repudiation at source and at destination.

#### Claim 10

Smart contract functions are triggered using signed transactions.

#### Proof

The use of smart contracts for each user of the system provides the service of non-repudiation at source and at destination, by the fact that every transaction is publicly stored on the blockchain together with the sending and receiving Ethereum address information. The signature on the transactions gives support to the non-repudiation features. $\square$

**6.**: **Self-Sovereignty**

#### Proposition 6

The owner users have the full control on their digital COVID-19 certificates.

#### Claim 11

The protocol presented in this paper provides the self-sovereignty of the medical data thanks to the PRE service. The owner users have the full control over the digital COVID-19 certificates that owns and decides with whom they want to share them.

#### Proof

Only authorized users can manage and share digital COVID-19 certificate. By the introduction of onlyOwner^4^ Solidity modifiers, the functions of the smart contracts can only be executed by the intended owners. Thus, only the users that owns the appropriate Ethereum addresses can sign transactions in order to have control over their owned certificates. Moreover, some functions can only be executed by a specific actor of the system, by the use of the onlyUser, onlyLab and onlyWHO modifiers. Besides, the PRE service is taking care of the cryptographic operations but it neither can get access to the content of the certificates nor tamper or steal the users certificates [42]. Consequently, no personal information about the users is published in the system. $\square$

**7.**: **Immutability**

#### Proposition 7

The system provides the immutability property to the digital COVID-19 certificates.

#### Claim 12

The records on the blockchain, once mined are fixed and immutable.

#### Proof

Blockchain blocks are identified and linked with their data hash, if the block contained data is modified or manipulated would change its hash and will invalidate the chain.

$$\square $$

#### Claim 13

The files stored in the IPFS system cannot be altered.

#### Proof

The content of an IPFS file cannot be changed without altering the content identifier (CID) of the file. Thanks to the IPFS content addressing if a digital COVID-19 certificate is altered would generate a new CID, and this would not be pointed from the blockchain register. $\square$

**8.**: **Auditability**

#### Proposition 8

Any interested party can verify the existence of a digital COVID-19 certificate without breaching confidentiality.

#### Claim 14

It is possible to prove that a digital COVID-19 certificate of the system exists at any given time and it will not change.

#### Proof

Thanks to the special blockchain characteristics: persistency, immutability and time stamping, the existence of a specific digital COVID-19 certificate can be verified at any time. $\square$

**9.**: **Authorisation**

#### Proposition 9

The digital COVID-19 certificates only can be accessed with the digital COVID-19 certificate owner’s authorisation.

#### Claim 15

Only registered users, trusted entities and laboratories can perform the different protocol transactions.

#### Proof

The system smart contracts code uses different modifiers to restrict transactions from being executed only from authorised Ethereum addresses that are registered.

$$\square $$

#### Claim 16

The system users and trusted entities can only decrypt certificates if they have both the WHO and the owner user authorisation.

**Fig. 13:**
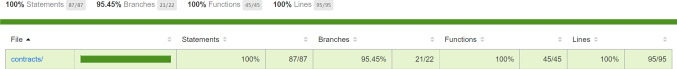
Code coverage report

#### Proof

The users that want to verify a digital COVID-19 certificate can only access it if, first of all, the WHO has accepted the request, and second, the owner user has also accepted it, generating the re-encrypted key for the trusted entity or user applicant. As regard to trusted entities, they can only access a digital COVID-19 certificate if they are registered as trusted entity and the certificate owner accept the access request from the trusted entity, generating the re-encrypted key. $\square$

**10.**: **Traceability**

#### Proposition 10

The generation and validation of COVID-19 digital certificates can be reviewed by reproducing the sequence of actions.

#### Claim 17

Thanks to the blockchain signed transactions, the system provides the possibility to track the different actions performed to issue or validate a digital COVID-19 certificate. It also allows to know who has performed each action.

#### Proof

All the system actions are performed through a smart contract transaction that contains the different Ethereum addresses that are involved, the action performed and the non-confidential parameters that are transmitted. This information would be useful in the circumstances where it may be necessary to track, at any time, the actions performed and the actors involved.$\square$

### Vulnerability analysis of the code

When implementing a protocol, it is important to test it to eliminate any possible vulnerabilities [44]. For this purpose, the implementation of smart contracts has been tested using the *Hardhat* *Code Coverage* plug-in, in conjunction with the Hardhat network, in order to detect weaknesses, risks and possible attacks that may compromise the security of the protocol.

The Code Coverage^5^ plug-in requires the generation of a Solidity code test using the Mocha and Chai libraries. If the tests run all the code of the different smart contracts (statements, functions, breaches and lines) defined, and the results are as expected, the coverage report indicates that the code is unlikely to contain unexpected bugs.

The code of our three smart contracts has passed the test, designed to check the correct performance of the different phases that make up the protocol, as well as to verify the expected values of important variables and the detection of important security errors. Therefore, with the test all functions have been verified automatically, as can be seen in the code coverage Istanbul report, Fig. 13. Only a few breaches (4.55%) could not be verified by the Hardhat code coverage plug-in. It is due to the onlyLab() modifier of the users’ smart contract. Therefore, we have checked it manually.

**Table 1:** Table representing the cost of executing the transactions of the main functions of the implementation

Function	Gas (weis)	USD (1Gwei)	USD (10Gwei)
Regulatory authority	4,913,478	9.04	90.42
User registration	2,687,489	4.95	49.45
Laboratory addition	416,236	0.77	7.66
Laboratory removal	24,834	0.05	0.47
Certificate generation	312,792	0.58	5.76
Entity management	36,099	0.07	0.66
Certificate sharing	868,014	1.6	15.97

## Performance analysis

In order to obtain a key point for a results analysis of the decentralised application implemented, a performance analysis has been carried out, studying the waiting time and the cost of executing the different transactions carried out.

The transactions carried out on the Ethereum blockchain network, where the execution of the smart contracts code is performed on the EVM, have a computational cost, which translates into a certain economic cost. In addition, in parallel to this cost, a waiting time is introduced, determined by the time required for the blockchain network to publish a new block.

The values obtained during the execution of the code introduced in Sect. 7 using the Rinkeby network, the private test network of the Ethereum blockchain, are introduced below. It should be noted that because it is a test network, the values obtained in this analysis may not be 100% accurate in relation to the costs that would be obtained with the deployment on the Ethereum blockchain.

Specifically, the set of the eight most important functions of the implementation have been chosen for the performance analysis.

### Execution costs

The cost of transactions is calculated in gas, which uses the unit Gwei. On the price of the transactions, an extra value can be introduced by the users, so that this can be obtained as a profit for the miners, therefore with the introduction of this extra value the transactions are verified more quickly.

In the analysis, a difference of 10 units of gas (10Gwei) over the transaction price (1Gwei) is considered in order to get the transaction confirmed by the miners in a shortest time.

The following table (Table 1) presents the results obtained, specifically the first column presents the function analysed, the second column represents the cost of the transaction in units of gas. And the last two columns represent the equivalent price in US Dollars, taking into account the relationship between the two currencies on 27 June 2021, when the price of Ether was at $1840.36, for the gas cost of 1Gwei and 10Gwei, respectively.

From Table 1 it can be seen that the deployment of the regulatory authority’s smart contract is the transaction with the highest cost, followed by the registration of new users to the system, a function which, in addition to storing the new user’s data in the regulatory authority’s smart contract, also deploys the smart contract owned by the user who executes the function. This high cost is mainly due to the need to introduce all the bytecode that forms the smart contract within the EVM.

The deployment of the smart contract of new laboratories is carried out by executing the function of adding a laboratory to the system. The deployment of this smart contract has a lower cost because the code is much simpler.

As far as the transaction cost of the main functions that make up the implementation is concerned, compared to the deployment of smart contracts, it has a much lower cost. Starting with the execution of the removal of a laboratory together with the addition and removal of an entity to the system are the main functions of the implementation that have a lower cost per transaction, due to the fact that they involve the publication of very few parameters in the network.

On the other hand, the functions of generating a digital COVID-19 certificate and sharing it with a trusted entity or other users of the system result in a higher cost per transaction, because all necessary parameters must be entered for the correct decryption by the PRE.

Therefore, as can be seen from the values obtained in this analysis, Table 1, the deployment and execution of the smart contracts implementation is relatively cost-effective.

### Delay costs

The Rinkeby test network indicates on its official website^6^ that the average waiting time for the publication of a new block on the network is approximately 15 s. Therefore, from the moment the user accepts the execution of a transaction and its introduction into the network, a waiting time is defined.

**Table 2:** Representative table of the waiting time for the confirmation of transactions

Function	Time (s)
User registration	17.66
Laboratory addition	18.61
Laboratory removal	18.89
Certificate generation	16.1
Entity management	19.51
Certificate sharing	18.93

The previous table (Table 2) shows the average waiting time for the confirmation of transactions by the Rinkeby network. Specifically, each action has been executed 10 times to obtain a stable value.

As can be seen in Table 2, the waiting time for the confirmation of the different transactions is very similar in all the main functions analysed, but it should be noted that these values are only indicative, as the waiting time is strongly dependent on the congestion experienced by the network at the time of the transaction. But taking into account possible network congestion, the waiting time will always be much shorter than with the use of other systems.

## Conclusions and future work

The management of the situation caused by COVID-19 pandemic requires the use of certificates that prove some personal circumstances, as vaccination or test results. Nowadays, several solutions have been presented and some of them are currently used. Some problems have arose from the use of these solutions. In some countries the certificates have been counterfeited, and other solutions do not take into account the high privacy requirements of the medical data and its sovereign identity. Moreover, all the parties involved in the procedure must be managed in a secure way to avoid attacks to the system.

In this paper, a blockchain-based protocol for COVID-19 digital certificate management system using a proxy re-encryption service has been presented, which provides high privacy, authenticity and self-sovereignty of data.

We present a useful solution for the management of the pandemic, through a fully decentralised implementation and ensuring the confidentiality of the user’s data, a very important feature as it deals with health data. Specifically, the proposed protocol fulfils the goals presented in Sect. 3: supervision of issuers and verifiers, encryption of all the digital COVID-19 certificate data, data sovereignty an fully implementation, analysis and testing of the protocol.

The fundamental parts that define the designed protocol are the use of the proxy re-encryption service to achieve data confidentiality and the self-management of the data by its owners. The use of the IPFS distributed storage system to store the digital COVID-19 certificates, encrypted by the PRE service, which provides permanent access to all certificates, and a regulatory authority, in charge of the system security management, that verifies the digital COVID-19 certificate issuers and verifiers.

The proposed protocol can be used for the management of digital COVID-19 certificates and, following the same criteria, it can be used for the management of other viruses or diseases and also could even be extended to the management of medical data of any kind, such as medical records or test results.

A set of possible future improvements to be made to the design and implementation of the protocol presented are:

1.: To achieve more global implementation and use the threshold encryption scheme. In the future we will implement a network of distributed PREs that will be used by the different users of the network. And also in this point it would be interesting to split the coding part of the PRE service and introduce the public methods to the different servers that conforms the network.
2.: The proposal allows the regulatory authority to filter the requests of sharing digital COVID-19 certificates between users and accepting or denying them. But in order to achieve a more decentralised system and to improve the use of self-sovereign identities, it could be useful change it to achieve that the requests always reach the final recipient, but the regulatory authority can send a notification to the user together with the request indicating that the requesting user has not been checked and could be a malicious user. And finally it would be the receiving user who end up making the decision whether to share the data or not.
3.: Introduce the use of QR codes to facilitate the handling from the point of view of the end users of the DApp.
4.: Conduct a study to reduce the cost of gas transactions, considering the possibility of deploying the set of smart contracts that make up the implementation in other block-chain platforms.
5.: Introduce of a metadata system that allows trusted entities, authorised by the user, to automatically request the re-encryption of the user digital COVID-19 certificates. Thus ensuring that the user does not have to participate in each access request.

## Data Availability

All data generated or analysed during this study are included in this published article.

## Footnotes

1 https://covid19.who.int/.

2 https://github.com/secomuib/HighlyPrivateManagementSystemForDigitalCOVID-19Certificates.

3 https://docs.ipfs.io/.

4 https://docs.soliditylang.org/en/v0.8.7/contracts.html.

5 https://hardhat.org/plugins/solidity-coverage.html.

6 https://www.rinkeby.io/#stats.
